# Fucose-Containing Sulfated Polysaccharides from Brown Seaweeds Inhibit Proliferation of Melanoma Cells and Induce Apoptosis by Activation of Caspase-3 *in Vitro*

**DOI:** 10.3390/md9122605

**Published:** 2011-12-13

**Authors:** Marcel Tutor Ale, Hiroko Maruyama, Hidekazu Tamauchi, Jørn D. Mikkelsen, Anne S. Meyer

**Affiliations:** 1 Center for Bioprocess Engineering, Department of Chemical and Biochemical Engineering, Technical University of Denmark (DTU), Soeltoft Plads Bldg. 229, 2800 Kgs. Lyngby, Denmark; Email: mta@kt.dtu.dk (M.T.A.); jdm@kt.dtu.dk (J.D.M.); 2 Department of Pathology, School of Allied Science, Kitasato University, Kitasato 1-15-1, Sagamihara, Kanagawa 252-0373, Japan; Email: maruyama@ahs.kitasato-u.ac.jp; 3 Department of Microbiology, School of Medicine, Kitasato University, Kitasato 1-15-1, Sagamihara, Kanagawa 252-0373, Japan; Email: hidetama@med.kitasato-u.ac.jp

**Keywords:** fucoidan, anti-tumor, sulfated polysaccharides, bio-activity, apoptosis, fucose

## Abstract

Fucose-containing sulfated polysaccharides (FCSPs) extracted from seaweeds, especially brown macro-algae, are known to possess essential bioactive properties, notably growth inhibitory effects on tumor cells. In this work, we conducted a series of *in vitro* studies to examine the influence of FCSPs products from *Sargassum* *henslowianum* C. Agardh (FSAR) and *Fucus vesiculosus* (FVES), respectively, on proliferation of melanoma B16 cells and to investigate the underlying apoptosis promoting mechanisms. Cell viability analysis showed that both FCSPs products, *i.e.*, FSAR and FVES, decreased the proliferation of the melanoma cells in a dose-response fashion, with FSAR being more potent at lower dosages, and FVES being relatively more anti-proliferative than FSAR at higher dosages. Flow cytometric analysis by Annexin V staining of the melanoma cells exposed to the FCSPs products confirmed that both FSAR and FVES induced apoptosis. The FCSPs-induced apoptosis was evidenced by loss of plasma membrane asymmetry and translocation of the cell membrane phospholipids and was accompanied by the activation of caspase-3. The FCSPs bioactivity is proposed to be attributable to distinct structural features of the FCSPs, particularly the presence of sulfated galactofucans (notably in *S.* *henslowianum*) and sulfated fucans (notably in *F. vesiculosus*). This study thus indicates that unfractionated FCSPs may exert bioactive effects on skin cancer cells via induction of apoptosis through cascades of reactions that involve activation of caspase-3.

## 1. Introduction

Fucose-containing sulfated polysaccharides (FCSPs) designate a group of diverse polysaccharides that can be extracted from brown seaweeds of the class Phaeophyceae. This seaweed class includes the order Fucales, in which seaweed species such as *Fucus* sp. and *Sargassum* sp. belong. The most studied FCSPs, originally called fucoidin, fucoidan or just fucans, have a backbone built of (1→3)-linked α-L-fucopyranosyl residues or of alternating (1→3)- and (1→4)-linked α-L-fucopyranosyl residues [1,2]. These fucopyranosyl residues may be substituted with short fucoside side chains or sulfate groups at C-2 or C-4, and may also carry other minor substitutions, e.g., acetate, xylose, mannose, glucuronic acid, galactose, or glucose [3,4,5]. Brown seaweed FCSPs also include sulfated galactofucans with backbones built of (1→6)-β-D-galacto- and/or (1→2)-β-D-mannopyranosyl units. In addition to sulfate these backbone residues may be substituted with fucosides, single fucose substitutions, and/or glucuronic acid, xylose or glucose substitutions [4]. Recently it has been understood that the compositional and structural features of FCSPs differ significantly among seaweed species and that these features are markedly influenced by the conditions used to extract them [3,6]. 

FCSPs of different degrees of purity and composition, extracted from brown seaweeds such as *Sargassum* sp. and *Fucus* sp., have been documented to have a wide range of biological activities including anticoagulant [7,8], antithrombotic [8], anti-inflammatory [9], anti-viral [10,11]; and notably anti-tumoral effects [8,12,13]. Unfractionated FCSPs have thus specifically been found to reduce cell proliferation of lung carcinoma and melanoma cells *in vitro*; to exert immunopotentiating effects in tumor bearing animals; and to activate natural killer cells in mice leading to increased anti-tumor effectiveness [13,14,15,16]. Kim *et al.* [17] applied a crude polysaccharide composed predominantly of sulfated fucose from *Fucus vesiculosus* to human colon cancer cells *in vitro*, and concluded that this crude brown seaweed polysaccharide extract can induce apoptosis, and provided data that suggested that the apoptosis was induced via activation of caspases. Moreover, commercially available crude FCSPs (“fucoidan”) extracted from *F. vesiculosus* have been reported to inhibit proliferation and induce apoptosis on human lymphoma HS-Sultan cells lines by activation of caspase-3 [18]. Recently, we have reported that crude FCSPs extracted from a *Sargassum* sp. and from *F. vesiculosus*, respectively, induce growth inhibition and apoptosis of melanoma B16 cells *in vitro* [13]. When injected intraperitoneally into mice over four days, these same unfractionated FCSPs were found to induce enhanced natural killer cells (NK cells) activity to result in specific lysis of YAC-1 cells (a murine T-lymphoma cell line sensitive to NK cells) [13]. Previous reports with human HS-Sultan cells and MCF-7 cells, respectively, have suggested that the FCSPs induced apoptosis initiation may take place via activation of caspase-3 and caspase-8 dependent pathways, respectively [18,19], but no firm evidence has been established regarding the exact mechanism responsible for the apoptotic action of the FCSPs. The objective of the present study was, therefore, to examine whether the anti-proliferative action and apoptosis of melanoma B16 cells induced by FCSPs derived from *Sargassum henslowianum* C. Agardh and *Fucus vesiculosus*, are accompanied by increased caspase-3 activity. We also wanted to evaluate whether any structural features of the FCSPs might be crucial for bioactivity. In this study, we present the different structural features of the FCSPs derived from *S. henslowianum* and *F. vesiculosus* as assessed by IR and ^1^H NMR spectroscopy and show that these FCSPs exert bioactive effects that inhibit the proliferation of melanoma B16 cells by apoptosis. We also show that the antiproliferative effects and the apoptosis are accompanied by activation of caspase-3.

## 2. Results

### 2.1. FCSPs Chemical Composition

The compositional analysis of the fucose-containing sulfated polysaccharide products from *S. henslowianum* C. Agardh (FSAR) and *F. vesiculosus* (FVES), respectively, showed that the FSAR product was mainly made up of uronic acid and fucose, with a significant level of sulfate, and minor amounts of other monosaccharides, mainly galactose and mannose (Table 1). The FVES product had a similar monosaccharide profile and a similar sulfation level, but the amounts of fucose, galactose and xylose were significantly higher than in FSAR; whereas the uronic acid and mannose levels were lower (Table 1). 

**Table 1:** Monosaccharide composition and sulfate content of the fucose-containing sulfated polysaccharides: *Sargassum henslowianum* C. Agardh (FSAR) derived from *S. henslowianum* C. Agardh and *Fucus vesiculosus* (FVES) derived from *F. vesiculosus*, respectively.

Samples	Monosaccharide Composition * in mg/g DW								
	Fuc **	Rha	Ara	Gal **	Glc	Xyl **	Man **	UA **	Sulfate
FSAR	31 ± 2	1.6 ± 0.1	0.2 ± 0.1	14 ± 1	4.2 ± 0.1	4.2 ± 0.3	5.7 ± 0.5	123 ± 7	384 ± 26
FVES	139 ± 5	2.0 ± 0.6	2.8 ± 0.2	28 ± 1	2.5 ± 1.8	13 ± 2	0.2 ± 0.4	19 ± 2	342 ± 45

* Monosaccharide composition: Fuc = fucose, Rha = rhamnose, Ara = arabinose, Gal = galactose, Glc = glucose, Xyl = xylose, Man = mannose, UA = uronic acid; ** Significantly different levels among FSAR and FVES at *P* ≤ 0.05, number of replicates = 4.

### 2.2. IR and ^1^H NMR Spectra of FCSPs

**Figure 1:**
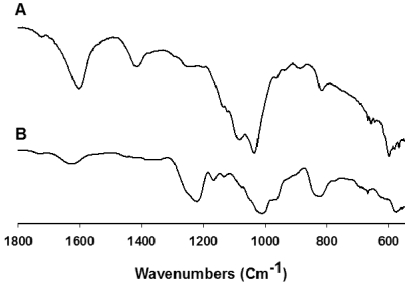
Infrared analysis of fucose-containing sulfated polysaccharides (FCSPs) from (**a**) *Sargassum* *henslowianum* C. Agardh (FSAR) and (**b**) *Fucus vesiculosus* (FVES) scanned between 1800 and 550 cm^−1^.

The FCSPs were analyzed to determine if their infrared absorption properties were similar to the previously reported fucoidan IR absorption data [2,20]. The spectra of the FSAR and FVES samples scanned between wavenumbers 4000 and 400 cm^−1^ both exhibited major absorption bands at around 3340 and 3420 cm^−1^ that were interpreted as being due to O–H stretching (data not shown). The IR spectra between 1800 and 500 cm^−1^ (Figure 1a,b) revealed small but distinct bands for both the samples at 1720 cm^−1^ which indicated the presence of *O*-acetyl groups [21], whereas the absorption bands at ~1610 to 1620 cm^−1^ (Figure 1a,b), most pronounced for the FSAR sample, indicated uronic acid [20]. The FSAR sample showed an intense IR band at around 1400–1470 cm^−1^ which could be attributable to scissoring vibration of CH_2_ (galactose, mannose) and asymmetric bending vibration of CH_3_ (fucose, *O*-acetyls) as suggested previously for absorption at around 1455 cm^−1^ by Synytsya *et al.* [22]. The absorption band at 1240 cm^−1^ observed for both samples, but being particularly prevalent for the FVES sample, was assigned as S=O stretching vibration, indicating the presence of esterified sulfate [20]. A similar absorption pattern around 820–840 cm^−1^ was observed for both FCSPs: The FSAR infrared spectrum showed an absorption band at 817 cm^−1^ (Figure 1a) whereas the FVES infrared spectrum displayed a broader absorption band at 838 cm^−1^ and a small shoulder of absorption at 822 cm^−1^ (Figure 1b). Since IR adsorption at 840 cm^−1^ has been reported to be due to sulfate groups at the axial C-4 position whereas sulfate groups at the equatorial C-2 and/or C-3 positions have been reported to give a small absorption at 820 cm^−1^ [2], the observed absorption bands at 820–840 cm^−1^ were interpreted as being indicative of sulfate groups. 

**Figure 2:**
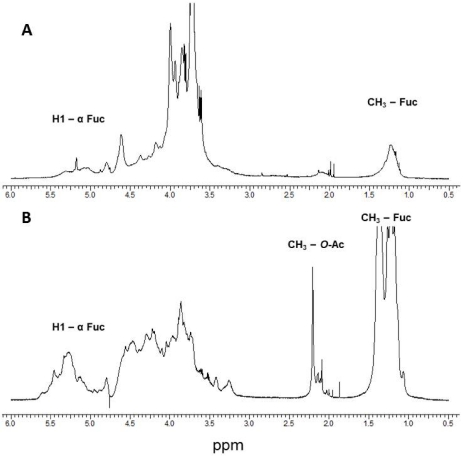
One-dimensional ^1^H NMR spectra of crude FCSPs from (**a**) *Sargassum henslowianum* C. Agardh (FSAR) and (**b**) *Fucus vesiculosus* (FVES) in D_2_O obtained using an INOVA 600 NMR spectrometer (Agilent Technologies, Tokyo, Japan).

The proton NMR spectra (^2^H_2_O) of the FSAR and the FVES samples were complex with broad signals and with several signals in the chemical shift of the envelope of anomeric signals at 5.0–5.5 ppm (Figure 2a,b). The presence of the signals at 5.0–5.5 ppm is consistent with the presence of α-L-fucopyranosyl [23]. The ^1^H-NMR spectra also both contained peaks at 1.1–1.3 ppm, with the signals for the FVES sample being particularly strong (Figure 2a,b). Previously, such high-field region signals at 1.1–1.3 ppm have been assigned to a C6 methyl proton group of L-fucopyranose [22] whereas several intense and narrow signals at 2.14–2.21 ppm have been attributed to CH_3_ protons of *O*-acetyl groups [5]. The narrow and intense signals at 5.10 and 5.18 ppm in the chemical shift of the envelope of the anomeric proton of FSAR (Figure 2a) were reported earlier and assigned to α3-linked and α3,4-linked L-fucopyranose residues for fucoidan from *Hizikia fusiformis* a.k.a *Sargassum fusiformis* [24]. The high field region signals 1.24 and 1.20 ppm (Figure 2a) were assigned to α3-linked 2-mono-*O*-sulfated and α3-linked unsulfated L-fucopyranose residues [21]. Moreover the intense signals at 4.37 and 3.99 ppm (Figure 2a) were assigned to the presence of 4-linked 2-mono-*O*-sulfated L-fucopyranose residues [21]. The independent signal, 4.61 ppm (Figure 2a) was assigned to a 3-linked D-galactopyranosyl residue when compared with the data of Farias *et al.* [25]. The FVES had an intense signal at 5.45 ppm (Figure 2b), which was assigned to α3-linked 2-mono-*O*-sulfated L-fucopyranose residues, whereas the signals at 5.40, 4.58 and 4.39 ppm (Figure 2b) were assigned to be due to di-sulfated residues, *i.e.*, α3-linked 2,4-di-*O*-sulfated L-fucopyranose residues [26]. Hence in general, the ^1^H NMR confirmed the anticipated FCSPs structures of the two samples. 

### 2.3. Anti-Proliferative Effects of the FCSPs

**Figure 3:**
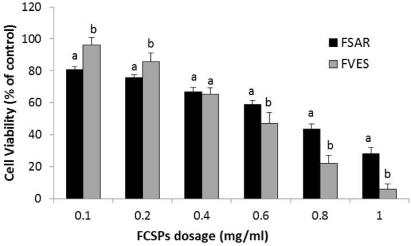
MTT based colorimetric assay of cell viability of melonama B16 cells after treatment for 24 h with different dosage levels of crude fucose-containing sulfated polysaccharides from *Sargassum* *henslowianum* C. Agardh (FSAR) and *Fucus vesiculosus* (FVES), respectively. Cell density was 6 × 10^4^ cells per well. ^a,b^ indicate statistically significantly different (*P* ≤ 0.05) cell viability levels after treatment with the two FCSP products at the same dosage level (mg/mL) (*n* = 4).

The viability of melanoma B16 cells treated with the FSAR and FVES products, respectively, was determined via measurement of cell proliferation using an MTT based colorimetric assay. Both FCSPs products (FSAR and FVES) decreased the viability of melanoma B16 cells in a dose-dependent fashion, after 24 h of incubation of 6 × 10^4^ cells density per well (Figure 3). In particular, a pronounced cell viability reduction was noticed after the addition of low levels, 0.1 mg/mL, of FSAR, producing a cell viability of ~80% of the control, and cell proliferation was halted gradually as the FCSPs dosage level increased (Figure 3) indicating moderate cytotoxicity. The FVES treated cells showed the same trend, but the FVES product generally induced a lower anti-proliferative effect than the FSAR product at the lower FCSP addition levels (*P* ≤ 0.05), but a significantly higher effect than FSAR (*P* ≤ 0.05) at the higher addition level, producing a drastic reduction of the proliferation of melanoma B16 cells leaving only ~6% of the cells viable at an FCSPs addition level of 1 mg/mL (Figure 3). The viability reduction pattern induced by the two FCSPs on the melanoma cells were in complete accord with previously published data [13]. 

### 2.4. Apoptosis of Melanoma Cells by FCSPs

**Figure 4:**
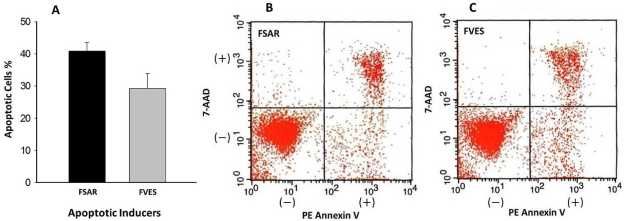
Flow cytometric analysis by Annexin V staining of Melanoma B16 cells treated for 24 h with 0.2 mg/mL crude fucose-containing sulfated polysaccharide (FCSP) products extracted from *S. henslowianum* C. Agardh (FSAR) and *F. vesiculosus* (FVES), respectively. (**a**) Apoptosis induced by FSAR (41 ± 3%, *n* = 2), FVES (30 ± 5%, *n* = 2); control 11.66 % (data not shown) (**b**) FSAR data and (**c**) FVES data for FACS scans of FCSP treated Melanoma 16 cells that were viable and not undergoing apoptosis (Annexin V^−^ and 7-AAD^−^); undergoing early apoptosis, with membrane integrity intact (Annexin V^+^and 7-AAD^−^); in the latest stage apoptosis and dead (Annexin V^+^ and 7-AAD^+^ ), respectively.

Programmed cell death or apoptosis is characterized by certain morphological cell changes such as loss of plasma membrane integrity in addition to internucleosomal DNA cleavage. One of the earliest apparent changes in cells undergoing apoptosis is the translocation of the cell membrane phospholipid phosphatidylserine from the inner to the outer leaflet of the plasma membrane. This change in the cell membrane is now recognized as an early, essential feature of apoptosis. The translocation exposes the phosphatidylserine to the external cellular environment and this is a feature which can be measured by exposing the cells to fluorochrome-conjugated phospholipid-binding proteins such as phycoerythin (PE)-labelled Annexin V (PE-Annexin V). Such staining with Annexin V is typically used in conjunction with a vital dye such as 7-amino-acticomycin (7-AAD) to identify early stages of apoptotic cells (Annexin V^+^, 7-AAD^−^) which accompany the later apoptosis stages (both Annexin V and 7-AAD are positive). Viable cells with intact membranes exclude 7-AAD, whereas the membranes of dead and damaged cells undergoing apoptosis are permeable to 7-AAD. Figure 4 shows the number of melanoma cells undergoing apoptosis (% relative to a control not exposed to FCSPs) and the flow cytometric scan data of Annexin V staining induced by exposure of the melanoma cells to 0.2 mg/mL of the seaweed FCSPs samples from *S. henslowianum* (FSAR) and *F. vesiculosus* (FVES), respectively. Both FCSPs products induced significant apoptosis of the melanoma cells: The FSAR product appeared to induce a more potent apoptotic effect than the FVES product (Figure 4a) since the relative number of melanoma cells undergoing apoptosis (% relative to a control sample not exposed to FCSPs) induced by the FSAR sample was significantly higher (41 ± 3%) than the apoptotic effect of the FVES exposure (30 ± 5%). The data corresponded to the fluorescence-activated cell sorting (FACS) scan showing the accumulation of intense dots-color in cells that underwent the latest apoptosis stage (Figure 4b,c: both Annexin V and 7-AAD positive). The FVES sample induced more early apoptosis (Figure 4c than the FSAR, as characterized by the build-up of disperse dots-color (Annexin V^+^ and 7-AAD^−^) indicating loss of plasma membrane asymmetry. The data were in accordance with the anti-proliferative effects of the FCSPs treatments (Figure 3).

### 2.5. FCSPs Activation of Caspase-3

**Figure 5:**
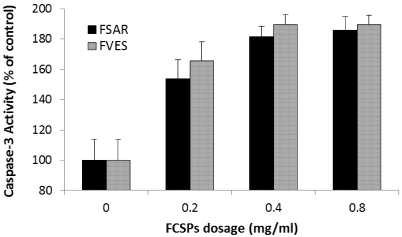
Activation of caspase-3 after treatment of melanoma B16 cells with different dosages of FCSPs from *S. henslowianum* (FSAR) and *F. vesiculosus* (FVES). For each dosage treatment the caspase-3 activity was assayed on a cytosolic extract of melanoma B16 cells with a DEVD-pNA substrate (contact at 37 °C for 1 h) and spectrophotometric detection by measuring the absorbance at 405 nm (*n* = 2).

In general, activation of caspase-3 initiates apoptosis in mammalian cells. The caspase-3 colorimetric assay employed in the present study is based on spectrophotometric detection of the chromophore p-nitroaniline (pNA) after cleavage of the pNA-labeled substrate DEVD-pNA. The activity of caspase-3 was augmented after treatment of the melanoma cells for 24 h with the FCSPs from *S. henslowianum* (FSAR) and *F. vesiculosus* (FVES) (Figure 5). The recorded caspase-3 activity increased significantly in a dose-dependent manner in response to the FCSPs treatment dosage (0–0.8 mg/mL), *i.e.*, from 100% of control at 0, to ~180% of the control response at 0.8 mg/mL (*P* ≤ 0.05) (Figure 5). No significant differences in the responses induced by the two types of FCSPs were recorded within the individual dosages of the FSAR and FVES treatments (Figure 5). The same trend of caspase-3 activity was observed in a 48 h treatment of melanoma cells with the FSAR and FVES samples (data not shown).

## 3. Discussion

The incidence of melanoma skin cancer has risen dramatically over the past few decades [27]. Because of the significant risk and undesirable effects of known cancer therapeutic strategies, many studies have evaluated the possible protective effects of bioactive compounds of natural origin. Fucose-containing sulfated polysaccharides (FCSPs) derived from naturally grown brown seaweeds by aqueous extraction have been shown to exert potentially beneficial bioactivities, including immuno-modulatory, anti-inflammatory and anti-tumorigenic effects. In keeping its natural properties, FCSPs must be extracted from brown seaweeds by use of a mild processing treatment and a minimal number of extraction steps.

Brown seaweeds constitute a part of the conventional diet in several Asian countries, especially in Japan, and in a Japanese cohort study the intake of seaweeds has been associated with lower mortality from all chronic diseases including cancer [28]. It has recently been demonstrated that FCSPs from brown seaweeds exert growth inhibitory activity on certain cancer cell lines *in vitro* [13,17,18]. Incorporation of brown seaweeds into animal diets has also revealed cancer inhibitory effects with no direct lethal consequences [29,30]. Natural FCSPs from brown seaweeds may therefore have significant potential as protective agents to control or prevent skin cancer provided that the FCSPs do indeed exert cancer-preventive effects. 

In this study, in accordance with previous data [13], we found that unfractionated FCSPs, *i.e.*, FSAR and FVES, extracted from the brown seaweeds *S. henslowianum* and *F. vesiculosus*, respectively, were composed of fucose, galactose, xylose, mannose and glucuronic acid, and showed that the fucose, galactose and glucuronic acid contents differed significantly among the two FCSPs products, but that their sulfate contents were similar (Table 1).

We also found both distinct differences and several similarities in the structural make-up of these FCPSs by use of FTIR and ^1^H NMR spectroscopy. The FT-IR analyses thus corroborated the presence of sulfate groups in both the FSAR and the FVES sample (Figure 1). The IR spectra indicated that the sulfate substitutions of the FCSPs extracted from the *Sargassum* sp. (FSAR) were located in the equatorial C-2 and/or C-3 positions as depicted by absorption bands at 817 cm^−1^. This finding was in agreement with data reported for fucoidan fractions isolated from *Sargassum stenophyllum* [4]. However, Duarte *et al.* [4] also reported that two other saccharide fractions from *S. stenophyllum* had an absorption band at 837 cm^−1^ indicating sulfate groups at the C-4 positions of the structural monosaccharides [4]. The spectra of the FCSPs from *F. vesiculosus* (FVES) displayed an absorption band at 838 cm^−1^ with a small shoulder at ~822 cm^−1^ indicating sulfate groups at both the C-4 and the C-2 position (Figure 1). This finding corresponds to previously reported ^1^H NMR data of FCSPs from *F. vesiculosus* that have indicated a typical structure of algal fucoidan consisting of α3-linked 2-mono-*O*-sulfated L-fucopyranose residues, and/or α3-linked 2,4-di-*O*-sulfated L-fucopyranose residues [2,26]. Small disparities in the IR spectra from different published reports can be due to factors such as sample handling and the FCSPs extraction procedure employed.

The present study also aimed at establishing whether crude FCSPs extracted from *Sargassum* *henslowianum* C. Agardh (FSAR) contained fucoidan-like structures composed of α-3-linked or/and α-3,4-linked L-fucopyranose residues. Even though signals consistent with the presence of α-L-fucopyranose entities were recorded (with ^1^H NMR signals at 5.10 and 5.18 ppm, Figure 2a), the probability that the FSAR may contain a cocktail of polysaccharides is likely. Hence, the ^1^H NMR spectra also showed that the FSAR sample contained 3-linked D-galactopyranose residues as indicated by an independent signal at 4.61 ppm (Figure 2a). β-(1→)3-linked galactopyranose residues are known to be a typical structural feature of seaweed polysaccharides, from e.g., *Laminaria angustata* var. *longissima*, *Botryocladia occidentalis* [25,31]. However, another possibility might be that the FSAR sample was not composed of a mixture of different types of polysaccharides but rather, that the sample consisted of one type of a highly complex hetero-polysaccharide as suggested by Duarte *et al.* [4] for the fucoidans from *Sargassum stenophyllum.* It can safely be said that the ^1^H NMR spectra of the FCSPs samples were complex and overlapping. It is therefore difficult to draw any definite conclusions about the detailed structural features and differences among the two FCSPs; the detailed elucidations of the definite structural details were also beyond the scope of this present study, but clearly deserve further investigation. Nonetheless, the data confirmed that the diversity, *i.e.*, the compositional and structural complexity of (potentially bioactive) algal fucose-containing sulfated polysaccharides, is much wider than originally believed.

The biological activities of the FCSPS against skin cancer cells were investigated *in vitro*, and the results revealed that both FSAR and FVES can exert anti-proliferative effects on melanoma B16 cells *in vitro*. The FSAR sample induced more significant reductions of the cell viability of melanoma cells than the FVES sample at low dosage levels (Figure 3). At higher dosages, the FSAR treatment still induced gradually more loss of cell viability, but the FVES had more potent anti-proliferative effects at higher dosages than FSAR which could indicate direct cell toxicity. The bioactivities of these FCSPs may be attributable to their distinct structural features, notably the level of sulfation (charge density), the distribution (e.g., random *versus* clustered) and bonding of the sulfate substitutions, as well as other specific structural features of the sulfated fucans and the sulfated galactofucan complexes. The sulfate groups of FVES were substituted at the C-2 and C-4 position of the fucose substituents, typical for fucoidan from *F. vesiculosus*, and consistent with previously published data indicating that the sulfate groups were substituted at C-2 of α3-linked L-fucopyranose residues in fucoidan from e.g., *Fucus evanescens* [21]. In contrast, the sulfate substitutions in the FSAR were interpreted to be mainly at the C-2 and/or C-3 positions of the monosaccharides according to the IR spectra (Figure 1); the observation of C-2 linked sulfate groups agreed with the data mentioned above for fucoidan from *Fucus* spp. [3,21], but is also in agreement with the proposition that the sulfate groups were substituted at C-2 on the 3-linked galactopyranose residues [25]. The possible presence of sulfated, 3-linked galactan in the structure of FSAR may contribute to the efficacy of FSAR to induce anti-proliferative effects as it has been reported that 2-*O*-sulfated 3-linked galactan is more bioactive than 2-*O*-sulfated 3-linked fucans and 3-*O*-sulfated 4-linked galactan [32,33,34]. 

The findings that the FCSPs induced apoptosis of the melanoma B16 cells *in vitro* were in agreement with recent reports [13,16,35], but the differential apoptotic efficacies, and the dose-response effects of differently structured FCSPs (Figure 4) have not been reported earlier. In particular, it is a novel finding that significantly different sulfated, polysaccharide structures from brown seaweeds—as evaluated in the present work—exert relatively similar apoptotic effects on melanoma cells. The results of this work thus indicate that not only the well-studied, classical type of FCSPs having a backbone made up of (1→3)-linked α-L-fucopyranosyl or of alternating (1→3)- and (1→4)-linked α-L-fucopyranosyl residues have potential tumor-preventing effecs, but also that the more complex sulfated fucose-rich galacto-mannans from *Sargassum* spp. exert promising cancer-preventive effects. The principal objective of this study was to assess whether any structural features of the FCSPs might be crucial for bioactivity, and the data suggest that the sulfate substitutions, and not necessarily only the fucose-backbone structure itself, confer this decisive bioactivity. It is however important to investigate whether other differently structured FCSPs may exert similar growth inhibitory and apoptosis inducing effects on cancer cells. 

**Figure 6:**
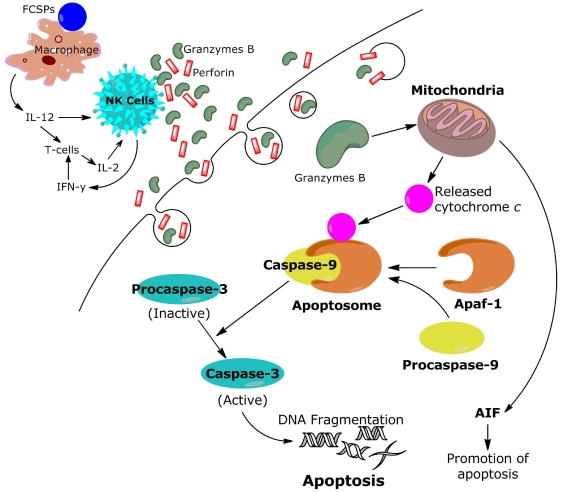
Proposed mechanism for inhibition of the proliferation of melanoma cells by FCSPs: Activation of macrophages via membrane receptors, which leads to the production of cytokines that enhance NK cell activation. Activated NK cells release Granzyme B and perforin through granule exocytosis into the space between NK cells and melanoma cells to initiate caspase cascades in melanoma cells. Assimilation of Granzyme B by the tumor cells is facilitated by perforin. Granzyme B then initiates apoptosis by triggering the release of mitochondrial cytochrome c and apoptosome formation leading to caspase-3 activation, which in turn translocates the nucleus causing DNA fragmentation—the distinct morphological change of cells by apoptosis [36,37].

In this work we noted that both FCSPs activate caspase-3 in a dosage-response fashion (Figure 5). These findings affirmed the results reported previously which have shown that FCSPs (“fucoidan”) from *F. vesiculosus* induce apoptosis in human lymphoma HS-Sultan cell lines and in HT-29 and HCT116 human colon cancer cells *in vitro*, and moreover that the exposure of these cells to the *F. vesiculosus* FCSPs appear to activate caspase-3 [17,18]. The *F. vesiculosus* FCSPs treatment was also shown to enhance mitochondrial membrane permeability of human colon cancer cells *in vitro*, and to induce cytochrome c and Smac/Diablo release from the mitochondria [17]. It has also been reported that pretreatment of HT-29 and HCT116 colon cancer cells with individual caspase-8 or caspase-9 inhibitors (Z-IETD-FMK and Z-LEHD-FMK, respectively) prior to fucoidan exposure reduced the levels of caspases, including caspase-3 [17]. It has likewise been shown that pretreatment of human lymphoma HS Sultan cells with a pan-caspase inhibitor, z-VAD-FMK, reduced fucoidan-induced apoptosis [18]. Hence, the available data support the proposition that fucoidan-induced apoptosis occurs through caspase activation pathways. The cascade mechanism by which the caspase-activation is presumed to take place via the mitochondria-mediated apoptotic pathway is illustrated in Figure 6.

Loss of plasma membrane is one of the earliest features of apoptosis and Annexin V staining can identify apoptosis at an early stage. However this assay does not distinguish between cells that have undergone apoptotic death *versus* those that have died as a result of a necrotic pathway, because in either case the dead cells will stain with both Annexin V and 7-AAD. Both early and later apoptosis stages were observed by the FACS scanning indicating that the FCSPs had a direct apoptotic effect on the melanoma B16 cells (*in vitro*) (Figure 4). The direct apoptotic action of the FCSPs was probably due to the interaction of the highly negative charge density of the FCSPs with the melanoma B16 cells (as a result of the sulfation). Recently, we reported that crude fucoidan from *Sargassum* sp. could trigger apoptosis indirectly by enhancing the activity of natural killer (NK) cells activity *in vivo* [13]. NK cells produce immunologically important cytokines, notably IFN-γ, which can promote the activation of T-cells to produce interleukin-2 and -12 that in turn further enhance the NK cell activation [14,36]. 

The apoptosis induced by FCSPS via the activation of caspase-3 was reported previously to be mediated through a mitochondrial pathway [17,18,19,38]. However, it remains to be determined whether differences in FCSPs structures will influence the apoptotic mechanism, including the mitochondrial pathway apoptosis cascade. The route of the mitochondrially dependent apoptotic pathway is the release of apoptosis-inducing factor (AIF) and cytochrome *c* from the inner mitochondrial membrane into the cytosol. Cytochrome *c* interacts with Apaf-1 (apoptotic protease activating factor 1) and procaspase-9 to form the active apoptosome. The apoptosome then initiates the cleavage of procaspase-3, producing active caspase-3, which initiates the execution phase of apoptosis by proteolysis of substances whose cleavage commits the cell to apoptosis [39] (Figure 6). The influence of the different FCSPs structures on the mitochondrial membrane permeability and electric potential requires further study. We hope in the future to investigate the bioactivity and mechanism of FCSPs on certain degenerative diseases *in vivo* and to further elucidate specific molecular targets of FSCPs for inhibition of cancer cells.

## 4. Experimental Section

### 4.1. Chemicals

Dried *S. henslowianum* C. Agardh was obtained from Viet Delta Ltd. (Ho Chi Menh, Vietnam) and the Fucose-containing sulfated polysaccharides (FCSPs) from the *S. henslowianum* (FSAR) were extracted in our laboratory (see below). Crude fucoidan from *F. vesiculosus* (FVES) was obtained from Sigma-Aldrich (Steinheim, Germany); according to the product description the FVES had been prepared from *F. vesiculosus* via the extraction method described by Black and Dewar [40]. Hydrochloric acid (37%), D-glucose and D-xylose were purchased from Merck (Darmstadt, Germany). Trifluoracetic acid (99%, TFA), trichloroacetic acid (99%, TCA), CaCl_2_, Na_2_SO_4_, BaCl_2_, arabinose, rhamnose, D-galactose and L-fucose were from Sigma–Aldrich Co. (Steinheim, Germany). Agarose D-2 was obtained from Hispanagar (Burgos, Spain). Caspase-3 colorimetric assay kit was obtained from Biovision, Inc. (Mountain View, CA. USA). Minimal essential medium eagle (MEM-eagle) cell culture media was purchased from Sigma–Aldrich Co. (Steinheim, Germany); foetal bovine serum (FBS) was from Flow Laboratories (North Ryde, N.S.W., Australia); streptomycin–penicillin and Trypan Blue were from Gibco (Canada). Cell Proliferation Kit 1 was obtained from Roche Applied Science, Germany. The PE Annexin V Apoptosis Detection Kit 1 was obtained from BD Biosciences (Franklin Lakes, NJ, USA). All chemicals used were analytical grade.

### 4.2. Extraction of FCSPs from *S. henslowianum* C. Agardh

The *Sargassum* FCSP product (FSAR) used was extracted from *S. henslowianum* C. Agardh by use of an optimized single-step extraction procedure described previously [6]. Briefly, the dried *S. henslowianum* seaweed was ground and sieved to pass through a 500 µm sieve and 100 g of dried ground seaweed was extracted in 2 L of 0.03 M HCl with continuous stirring at 200 rpm for 4 h at 90 °C water bath (Julabo, Germany). The suspended seaweed was filtered, and the extract was precipitated using 60% ethanol, the precipitate collected after centrifugation at 10,600 rpm for 10 min (Sigma Laboratory Centrifuge 4K15, VWR, Denmark), and the resulting pellet was freeze dried. This freeze dried pellet constituted the fucose-containing sulfated polysaccharides (FSAR). 

### 4.3. Acid Hydrolysis and FCSPs Composition Analysis

The freeze dried FSAR and FVES samples (20 mg) were hydrolyzed separately in 2 M TFA (final concentration) at 121 °C for 2 h, then the hydrolyzed mixture were freeze dried at −57 °C (Heto Lyolab 3000, England). Each dried powder sample was resolubilized in doubly distilled water and centrifuged at 10,000 rpm for 10 min to collect the supernatant (Sigma Laboratory Centrifuge 4K15, VWR, Denmark). Each supernatant was filtered through a 0.2 µm syringe tip filter (SUN-Sri, Rockwood, TN) prior to injection into the HPAEC-PAD for monosaccharide analysis [41]. Analysis of sulfate content was done according to the method described by Jackson and McCandless [42]. 

### 4.4. ^1^H NMR and FTIR Spectroscopy

The ^1^H NMR spectra were obtained using an INOVA 600 NMR spectrometer (Agilent Technologies Japan, Ltd., Tokyo Japan) equipped with a ^1^H[^15^N-^31^P] pulse field gradient indirect-detecting probe. Standard pulse sequences were used in all operations. The ^1^H chemical shift (δH) was referenced to HOD (δH 4.76 ppm, ^2^H_2_O). The ^1^H NMR spectrum was assigned through the ^1^H–^1^H decoupling technique. An NMR spectrum of L-fucose was utilized as a reference for chemical shift assignment. The lyophilized FCSPs powders were dissolved in deuterium oxide (^2^H_2_O) and evaporated to exchange the unstable ^1^H with ^2^H. The evaporation and dissolution step was repeated five times, and the samples (10 mg) were finally dissolved in 0.75 mL ^2^H_2_O and then subjected to NMR spectroscopy. The IR spectra were obtained using a Spectrum One FT-IR spectrometer (Perkin Elmer, Waltham, MA, USA) equipped with universal attenuated total reflectance (UATR) accessories. Analysis of each of the FSAR and FVES powders, ~1 mg of each, was done using diffuse reflectance infrared transform spectroscopy (DRIFTS) and the spectrum was evaluated by Perkin Elmer Spectrum software version 5 (Perkin Elmer, Waltham, MA, USA). 

### 4.5. Cell Culture and Anti-Proliferative Assay

Melanoma B16 cells (MC) were grown in MEM eagle medium supplemented with 10% (v/v) heat inactivated FBS, 1% (w/v) streptomycin–penicillin and 1% (v/v) of 200 mM L-glutamine at 37 °C under 5% CO_2_. Monolayer cultivation was carried out by adding 100 µL of the cell-MEM-FBS mixture into separate wells in 96-flat well plates at a density of 6 × 10^4^ cells per well followed by incubation for 24 h in 5% CO_2_ at 37 °C. For the anti-proliferation assay the medium was removed after the 24 h of monolayer cell cultivation and replaced with 100 µL of MEM medium containing 2% FBS and varying concentrations (0.1–1.0 mg/mL) of the crude FCSPs, *i.e.*, FSAR and FVES, respectively, and the mixtures were then incubated for 24 h. Quantification of cell proliferation was carried out using a tetrazolium salt (MTT (3-(4,5-dimethyl-thiazolyl-2)-2,5-diphenyltetrazolium bromide)) based colorimetric assay following the protocol supplied with the Cell Proliferation Kit 1 (Roche Applied Science, Germany). Briefly, 20 µL MTT solution (5 mg/mL) was added to the cell cultures after the 24 h of incubation with the FCSPs, and the cell cultures were then re-incubated for 4 h. Finally, 100 µL of stabilization solution was added to each well and the plates were incubated overnight at 37 °C under 5% CO_2_. Absorbance was measured using an Elisa reader at 550–690 nm.

### 4.6. Cell Culture and Caspase-3 Assay

Melanoma B16 cells (MC) were grown in MEM eagle culture medium supplemented with 10% (v/v) heat inactivated FBS, 1% (w/v) streptomycin–penicillin and 1% (v/v) of 200 mM L-glutamine maintained at 37 °C under 5% CO_2_. For the caspase-3 assay, monolayer cultivation was carried out in a petri dish (60 × 15 mm) by adding 5 mL culture medium containing melanoma cells at a density of 1 × 10^5^ per mL and varying concentrations (0.2, 0.4 and 0.8 mg/mL) of the FSAR and FVES, respectively. The mixtures were then incubated for 24 and 48 h in 5% CO_2_ at 37 °C. The caspase-3 assay was performed according to the protocol supplied with the assay kit (Biovision Inc., Mountain View, CA, USA) used to assay the activity of caspases that recognize the amino acid sequence DEVD. The assay was based on spectrophotometric detection of the chromophore *p*-nitroaniline (pNA) after cleavage from the labeled substrate DEVD-pNA. Concisely, the melanoma B16 cells exposed to FSAR and FVES, respectively, were harvested and resuspended in 50 µL of cell lysis buffer and incubated on ice for 10 min. and the mixture centrifuged for 1 min (14,000 × *g*, 4 °C). Each supernatant was then transferred to a fresh tube, and reaction buffer (50 µL) and 4 mM DEVD-pNA substrate (5 µL) were added, and this reaction mixture was then incubated at 37 °C for 1 h. Absorbance of pNA light emission was quantified using a microtiter plate reader at 405 nm.

### 4.7. Apoptosis Assay by Fluorescence-Activated Cell Sorting (FACS)

After 24 h of monolayer cultivation of melanoma B16 cells with 0.2 mg/mL of FSAR or FVES, and no FCSPs addition as control, the culture medium was removed, and the cells harvested by addition of 1 mL Trypsin-EDTA. The harvested cells were washed twice with 0.1 M PBS and then resuspended in binding buffer according to the protocol for the Annexin V Apoptosis Detection Kit I (BD Biosciences, Franklin Lakes, NJ, USA). 100 µL of this solution at 1 × 10^5^ cells was transferred to a culture tube and 5 µL of Annexin V and 5 µL of 7-amino-actinomycin (7-ADD) were added, and the mixture incubated at room temperature for 25 min. Then, 400 μL of binding buffer was added and the extent of apoptosis and staining pattern of the cells were tracked by flow cytometric analysis on a FACScan instrument (Becton Dickinson).

## 5. Conclusions

The tumor inhibitory bioactivity of fucose-containing sulfated polysaccharides (FCSPs) from *Sargassum* *henslowianum* C. Agardh (FSAR) and *F. vesiculosus* (FVES) was demonstrated through evaluation of inhibition of melanoma cell proliferation, activation of caspase-3, and apoptosis of melanoma B-16 cells *in vitro*. The structural traits of the FCSPs products were shown to be complex and to differ among the two FCSPs making it delicate to draw definite conclusions about structural effects and mechanisms. However, since the sulfate levels were relatively high as well as relatively similar among the two FCSPs, we propose that the bioactivity effects of the FSAR and FVES might be attributable to the sulfation (charge density), positioning and bonding of the sulfate substitutions in the FCSPs. The work clearly indicates that unfractionated fucose-containing sulfated polysaccharides from both *Sargassum* *henslowianum* C. Agardh and *Fucus vesiculosus* may have therapeutic potential as skin-cancer preventive agents. 
